# Snake Deltavirus Utilizes Envelope Proteins of Different Viruses To Generate Infectious Particles

**DOI:** 10.1128/mBio.03250-19

**Published:** 2020-03-17

**Authors:** Leonora Szirovicza, Udo Hetzel, Anja Kipar, Luis Martinez-Sobrido, Olli Vapalahti, Jussi Hepojoki

**Affiliations:** a University of Helsinki, Medicum, Department of Virology, Helsinki, Finland; b Institute of Veterinary Pathology, Vetsuisse Faculty, University of Zürich, Zürich, Switzerland; c Department of Veterinary Biosciences, Faculty of Veterinary Medicine, University of Helsinki, Helsinki, Finland; d Department of Microbiology and Immunology, University of Rochester, Rochester, New York, USA; e Helsinki University Hospital Laboratory, Helsinki, Finland; Indiana University Bloomington

**Keywords:** coinfection, deltavirus, hepatitis, virology, zoonotic infections

## Abstract

Satellite viruses, most commonly found in plants, rely on helper viruses to complete their replication cycle. The only known example of a human satellite virus is the hepatitis D virus (HDV), and it is generally thought to require hepatitis B virus (HBV) to form infectious particles. Until 2018, HDV was the sole representative of the genus *Deltavirus* and was thought to have evolved in humans, the only known HDV host. The subsequent identification of HDV-like agents in birds, snakes, fish, amphibians, and invertebrates indicated that the evolutionary history of deltaviruses is likely much longer than previously hypothesized. Interestingly, none of the HDV-like agents were found in coinfection with an HBV-like agent, suggesting that these viruses use different helper virus(es). Here we show, using snake deltavirus (SDeV), that HBV and hepadnaviruses represent only one example of helper viruses for deltaviruses. We cloned the SDeV genome into a mammalian expression plasmid, and by transfection could initiate SDeV replication in cultured snake and mammalian cell lines. By superinfecting persistently SDeV-infected cells with reptarenaviruses and hartmaniviruses, or by transfecting their surface proteins, we could induce production of infectious SDeV particles. Our findings indicate that deltaviruses can likely use a multitude of helper viruses or even viral glycoproteins to form infectious particles. This suggests that persistent infections, such as those caused by arenaviruses and orthohantaviruses used in this study, and recurrent infections would be beneficial for the spread of deltaviruses. It seems plausible that further human or animal disease associations with deltavirus infections will be identified in the future.

## Abstract (precis)

Deltaviruses need a coinfecting enveloped virus to produce infectious particles necessary for transmission to a new host. Hepatitis D virus (HDV), the only known deltavirus until 2018, has been found only in humans, and its coinfection with hepatitis B virus (HBV) is linked with fulminant hepatitis. The recent discovery of deltaviruses without a coinfecting HBV-like agent in several different taxa suggested that deltaviruses could employ coinfection by other enveloped viruses to complete their life cycle. In this report, we show that snake deltavirus (SDeV) efficiently utilizes coinfecting reptarena- and hartmaniviruses to form infectious particles. Furthermore, we demonstrate that cells expressing the envelope proteins of arenaviruses and orthohantaviruses produce infectious SDeV particles. As the envelope proteins are responsible for binding and infecting new host cells, our findings indicate that deltaviruses are likely not restricted in their tissue tropism, implying that they could be linked to animal or human diseases other than hepatitis.

## INTRODUCTION

Viroids found in higher plants are the smallest known infectious agents and are comprised of only circular RNA (1). After the discovery of viroids in 1971 (2), hepatitis D virus (HDV) was described in 1977 (3) as the first human pathogen with an RNA genome resembling viroids. Like viroids, the circular genome of HDV forms secondary structures by self-complementarity, although the genome of HDV is roughly four times bigger (4). Viroids also replicate by the rolling circle mechanism and possess ribozyme activity (4). However, unlike viroids, HDV encodes a functional protein, and it also requires hepatitis B virus (HBV) as a helper virus (4). Until recently, deltaviruses had been found only in humans. Then, in 2018, HDV-like sequences were reported from two nonhuman hosts (5, 6). The findings challenged the view on the origin and evolution of HDVs within their human host (7).

Before the discovery of novel deltaviruses (5, 6, 8), HDV was unique among animal viruses, and it formed the genus *Deltavirus* which has not been assigned to a family (9). The negative-sense single-stranded RNA genome of HDV is approximately 1.7 kb, circular, and highly self-complementary, and as a result of this, it forms unbranched rod-like structures (10, 11). During replication, both genomic and antigenomic viral RNA are found in the infected cells (12). The only conserved open reading frame (ORF) of HDV is in antigenomic orientation and encodes the hepatitis delta antigen (HDAg, used for HDV) (10). Both RNA strands possess ribozyme activity responsible for self-cleavage (13). The ribozyme was initially speculated to also mediate the ligation to form the circular genome (14), but later studies indicated involvement of host enzymes (15). As HDV encodes only HDAg, it cannot form infectious particles without a helper virus (16). The discovery of HDV in liver specimens of HBV-positive individuals directly associated HDV with HBV as a satellite virus (3). Later studies demonstrated the transmissible and pathogenic nature of HDAg (17) and that HDV relies on the envelope glycoproteins of HBV to form infectious particles (16). Although helper virus is required for producing infectious particles, the rolling circle replication (12) proceeds independently of the helper virus (18), as it is mediated by host RNA polymerase II (19). During the viral life cycle, two different forms of HDAg, small HDAg (S-HDAg) and large HDAg (L-HDAg), are produced (20). The HDAg ORF encodes the S-HDAg, and a base transition in the amber stop codon results in the elongation of the protein at the carboxy terminus by 19 additional amino acids, thus giving rise to the L-HDAg (21). The two antigen forms have highly diverse roles in the viral life cycle, e.g., S-HDAg promotes viral replication, while L-HDAg inhibits viral replication (20, 22). In fact, L-HDAg also suppresses the expression of HBV proteins and acts in the assembly of infectious particles (23).

Due to the symbiotic relationship with HBV, HDV infection is acquired either via coinfection with HBV or via superinfection of a chronically HBV-infected individual (24). The disease outcome varies greatly and depends on the mode of HDV infection (25). Coinfection often results in acute hepatitis, which tends to be self-limited, whereas superinfection can lead to a fulminant hepatitis which in many cases becomes chronic, ultimately leading to liver cirrhosis (26). In these patients, the risk of liver failure or development of subsequent hepatocellular carcinoma is high (26). However, the disease is also influenced by the HDV genotype (25); eight HDV genotypes are currently known (27). While HDV-1 occurs worldwide, the other genotypes have a specific geographic distribution (27). Interestingly, a very recent study reported HDV to be capable of producing infectious particles utilizing envelope glycoproteins of several viruses (28).

The report on discovery of a HDV-like agent in birds (5) prompted us to publish our observation of a similar, yet genetically distant, agent in snakes (6). Both the avian and snake deltaviruses (AvDV and SDeV, respectively) possess a negative-sense, highly self-complementary circular RNA genome, including ribozymes (5, 6). The findings complemented one another, since no hepadnaviral sequences were identified in the samples (5, 6). Moreover, we could demonstrate the presence of both viral RNA and snake delta antigen (SDAg) in several tissues, indicating that SDeV replication is not restricted to the liver (6), which likely holds true also for AvDV. In further support of the idea that the evolutionary path of HDV is much longer than initially envisioned, more HDV-like agents were identified in fish, amphibians, and invertebrates in early 2019 (8). Similarly to AvDV and SDeV, the newly found deltaviruses were not associated with hepadnavirus infection (8). The new findings have raised the question whether deltaviruses are indeed dependent on hepadnavirus coinfection or not. The authors of the report on AvDV identified sequences matching influenza A virus genome in the same samples (5); similarly, we could demonstrate replication of both reptarenaviruses and hartmaniviruses in the snakes with SDeV (6). These findings led us to hypothesize that deltaviruses have evolved to use persistent or recurrent infection-causing enveloped viruses as their helpers. The aim of our study was to experimentally demonstrate that SDeV can utilize the envelope glycoproteins of viruses other than hepadnaviruses to form infectious particles.

(The first version of this article was submitted to an online preprint archive [29]).

## RESULTS

### Isolation of SDeV from the brain of an infected snake.

**FIG 1:**
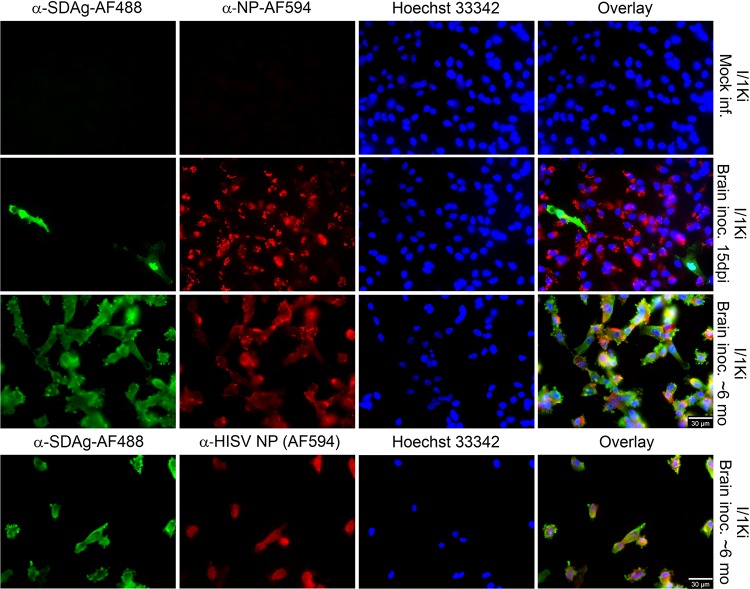
Isolation of SDeV from the brain of an infected snake using cultured boid kidney cells (I/1Ki). Mock-infected I/1Ki (top panels) and brain homogenate-inoculated I/1Ki cells (bottom panels) were stained for SDAg (anti-SDAg-AF488 [α-SDAg-AF488], left panels, green), reptarenavirus or hartmanivirus nucleoprotein [anti-NP-AF594 (α-NP-AF594) or α-HISV NP (AF594), middle panels, red], and Hoechst 33342 was used to visualize the nuclei. The panels on the right show an overlay of the three images. The images were taken at ×400 magnification using a Zeiss Axioplan 2 microscope. inf., infected; inoc., inoculated; dpi, days postinfection; mo, months.

We originally identified SDeV when performing a metatranscriptomic analysis of a brain sample from a snake with central nervous system signs (6). Subsequent reverse transcription-PCR (RT-PCR) screening demonstrated the presence of SDeV in multiple tissues, including liver and blood, and further metatranscriptomic analyses of blood and liver samples revealed no traces of an HBV-like virus; instead, we retrieved genomes of coinfecting reptarena- and hartmaniviruses. Since hepadnaviruses are hepatotrophic, we reasoned that successful isolation from the brain on cells other than liver cells would indicate that SDeV could utilize arenaviruses for the formation of infectious particles. We inoculated cultured boid kidney cells (I/1Ki) with the SDeV-infected brain homogenate and at 15 days postinfection (dpi) analyzed the cells by immunofluorescence (IF) staining. Affinity purification served to produce anti-SDAg and anti-NP (reptarenavirus nucleoprotein, the main antigen present in infected cells), which we directly labeled with Alexa Fluor 488 or Alexa Fluor 594 dyes (anti-SDAg-AF488, anti-SDAg-AF594, anti-NP-AF488, and anti-NP-AF594). Our studies show that the anti-NP antibody can be used to detect all reptarenavirus NPs thus far described (30–33). As demonstrated in Fig. 1, the reagents produce hardly any background and can be used for costaining of SDeV and reptarenaviruses. Figure 1 further demonstrates isolation of SDeV by inoculating I/1Ki cells with the infected brain homogenate. Titration of the supernatant collected at 7 dpi shows that the infected cells produce progeny virions on clean I/1Ki cells with titers reaching 4.0 × 10^3^ fluorescent focus-forming units/ml (fFFU/ml). To ascertain whether the number of SDAg-expressing cells would increase over time and whether SDeV could establish a persistent infection, we passaged the infected cells and analyzed them by IF staining at roughly 6 months after inoculation. The staining (Fig. 1) shows that the majority of cells are coinfected with SDeV, reptarenavirus, and hartmanivirus, suggesting a permanent infection with all three viruses.

### Transfection of cultured cells with SDeV constructs initiates replication of the virus.

**FIG 2:**
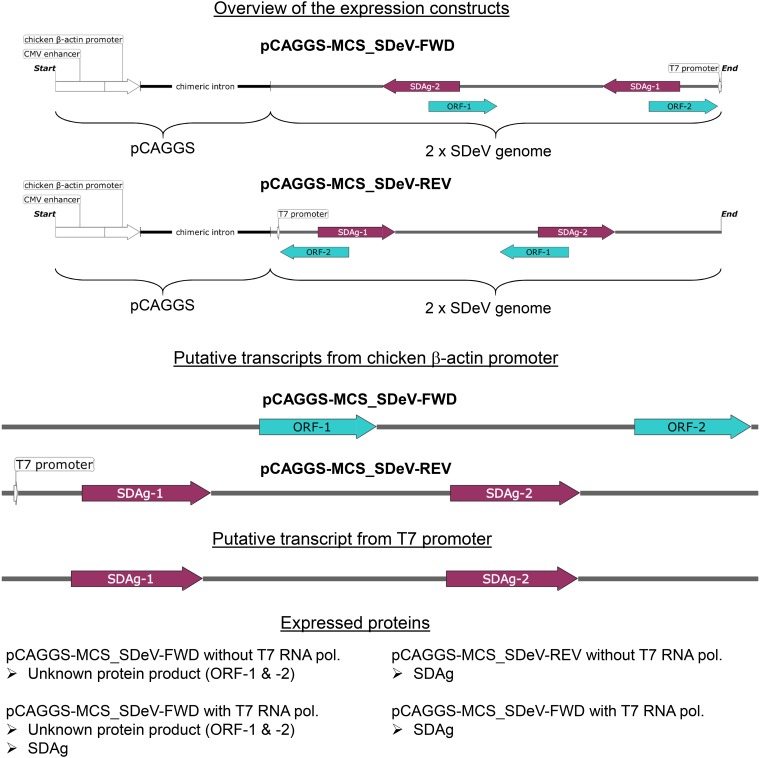
Inserts in the SDeV expression plasmids with putative transcripts and proteins produced. The inserts in pCAGGS-MCS_SDeV-FWD and pCAGGS-MCS_SDeV-REV with the cytomegalovirus (CMV) enhancer, chicken β-actin promoter, and chimeric intron from pCAGGS are schematically depicted at the top of the figure. Below are the putative transcripts produced under chicken β-actin promoter following transfection to cells, and transcripts produced in the presence of T7 RNA polymerase (or transcription from T7 promoter). The expressed proteins are listed at the bottom.

**FIG 3:**
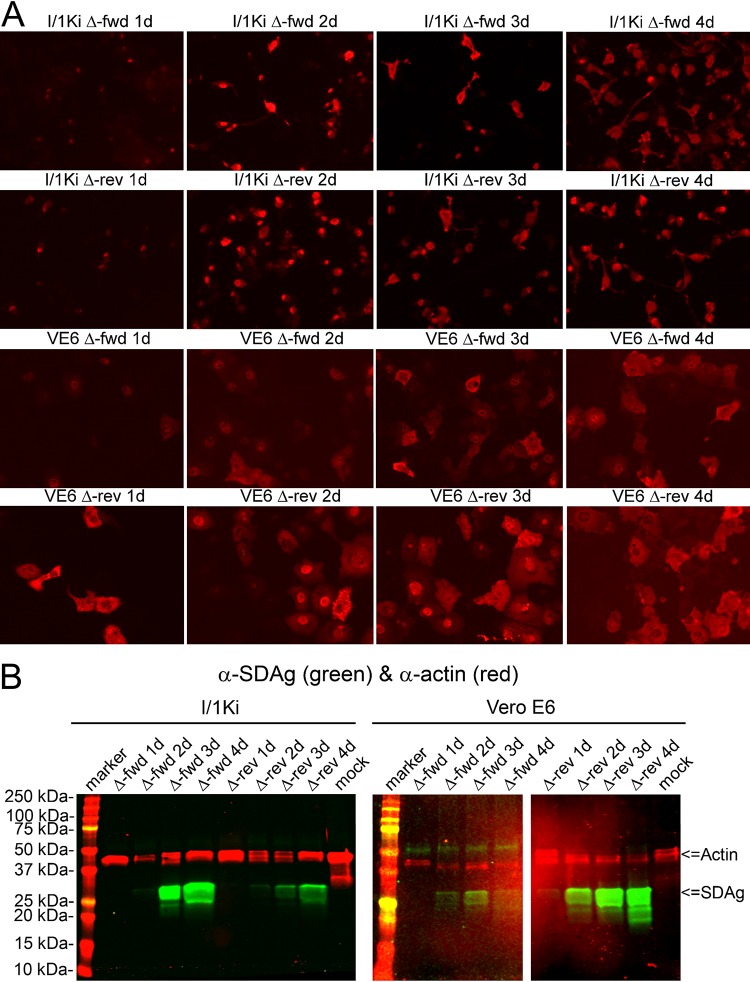
Transfection of I/1Ki and Vero E6 cells with pCAGGS-SDeV-FWD and pCAGGS-SDeV-REV constructs results in SDeV replication. (A) I/1Ki (top) and Vero E6 (bottom) cells transfected with Δ-fwd (pCAGGS-SDeV-FWD) and Δ-rev (pCAGGS-SDeV-REV) were stained for SDAg (anti-SDAg antiserum [1:7,500] and Alexa Fluor 594-labeled donkey anti-rabbit immunoglobulin [1:1,000]) at 1, 2, 3, and 4 days posttransfection (from left to right). The images were taken at ×400 magnification using a Zeiss Axioplan 2 microscope. (B) Western blot of I/1Ki (left panel) and Vero E6 (right panel) cell pellets at 1, 2, 3, and 4 days posttransfection with Δ-fwd and Δ-rev constructs. Precision Plus Protein Dual Color Standards (Bio-Rad) served as the marker, and the results were recorded using the Odyssey infrared imaging system (Li-Cor).

After successfully isolating SDeV in cell culture, we aimed to study SDeV replication without coinfecting viruses. As replication of HDV occurs via rolling circle replication (12), we generated expression constructs with multiple copies of the SDeV genome, an approach successfully applied in HDV reverse genetics (18). We ordered a synthetic gene comprising two copies of the SDeV genome and subcloned the insert in genomic (pCAGGS-SDeV-FWD [FWD stands for forward]) and antigenomic (pCAGGS-SDeV-REV [REV stands for reverse]) orientation into the mammalian expression vector pCAGGS/MCS (MCS stands for multiple cloning site). Because the expression of SDAg is considered essential for virus replication, we included a T7 promoter in antigenomic orientation to enable optional (i.e., when the T7 RNA polymerase is coexpressed) antigenomic transcription. The expression constructs are schematically presented in Fig. S1 in the supplemental material, and the inserts with putative transcripts produced from the constructs are shown in Fig. 2. Assuming that the snake cells do not initiate transcription at the T7 promoter, the pCAGGS-SDeV-FWD construct would produce SDAg only as a result of virus replication, whereas the pCAGGS-SDeV-REV construct would produce the SDAg without virus replication under the β-actin promoter of pCAGGS. To study SDAg expression, we transfected *Boa constrictor* (I/1Ki) and African green monkey (Vero E6) kidney cells and studied the cells by IF staining at 1, 2, 3, and 4 days posttransfection (dpt). As Fig. 3A demonstrates, SDAg can be detected from day 1 onwards with both constructs and cell lines. The fact that pCAGGS-SDeV-FWD transfection induces SDAg production suggests initiation of SDeV replication. Interestingly, at 1 and 2 dpt, the SDAg was predominantly found in the cytoplasm when expressed from the pCAGGS-SDeV-REV plasmid, whereas transfection with the pCAGGS-SDeV-FWD plasmid resulted in a predominantly nuclear localization of SDAg. At 4 dpt, however, SDAg was mostly detected in the cytoplasm for both constructs. The observed localization differences could be due to differences in transcription if the protein is produced under the β-actin promoter present in the plasmid (pCAGGS-SDeV-REV) compared to when the protein is expressed as a result of viral replication. The shuttle of the two HDAg forms between the nucleus and cytoplasm is mediated by posttranslational modifications (34), which could also be different in replication versus transcription from plasmid DNA. Furthermore, similarly to HDV, SDeV’s genomic and antigenomic RNA species could employ different cellular machineries for replication (35), thus adding a potential explanation to the observed differences. We also analyzed the transfected cells by Western blotting (WB) and could demonstrate an increasing amount of SDAg during the 4 days of transfection (Fig. 3B). The S-SDAg and L-SDAg have estimated molecular weights of 22.7 and 25.6 kDa, respectively (6). For a loading control, we probed the membranes with pan-actin antibody, which produced bands of expected sizes (around 42 kDa) in WB (Fig. 3B).

10.1128/mBio.03250-19.1 FIG S1 A schematic representation of the construction of the SDeV recombinant expression plasmids. The vector backbone (pCAGGS-MCS) is shown on the top left, and the final constructs are shown on the right. The inserts (bottom left) containing two copies of the SDeV genome were cloned into the MCS (multiple cloning site) as indicated by the red arrow. The inserts contain two copies of the SDAg (SDAg-1 and SDAg-2), open reading frame (ORF-1 and ORF-2) for an unknown protein product and T7 promoter. Blunt end cloning was used to obtain constructs (shown on the right) with the insert in genomic (pCAGGS-SDeV-FWD) or antigenomic (pCAGGS-SDeV-REV) orientation. Download FIG S1, TIF file, 1.5 MB. Copyright © 2020 Szirovicza et al. 2020 Szirovicza et al. This content is distributed under the terms of the Creative Commons Attribution 4.0 International license.

Because the RNA polymerase II machinery of some mammalian cell lines can use the T7 promoter for initiation of transcription (36, 37), we transfected I/1Ki cells with a pCAGGS construct bearing a synthetic UGV-1 small segment with hemagglutinin (HA)-tagged NP under the chicken β-actin promoter, and FLAG-tagged glycoprotein precursor (GPC) under the T7 promoter. To demonstrate that detectable GPC expression occurs only in the presence of T7 polymerase, we performed the transfections with and without a T7 polymerase-coding plasmid. This plasmid was extracted from BSR-T7/5 cells that are stably transfected with plasmid bearing T7 polymerase gene (38). WB of cells at 1, 2, and 3 dpt served to demonstrate that detectable GPC expression occurred only when the T7 polymerase plasmid was cotransfected (Fig. S2). The result further supports our interpretation that SDAg detected after transfection of snake cells with pCAGGS-SDeV-FWD is due to SDeV replication.

10.1128/mBio.03250-19.2 FIG S2 The snake cells do not utilize the T7 promoter. To demonstrate that SDAg detected in snake cells following pCAGGS-SDeV-FWD transfection is due to SDeV replication (as opposed to transcription from the antigenomic T7 promoter), we transfected I/1Ki cells with synthetic UGV-1 S segment bearing pCAGGS. (A) The synthetic S segment contains HA-tagged UGV-1 NP under chicken β-actin promoter (in pCAGGS), and FLAG-tagged UGV-1 GPC in antigenomic orientation under the T7 promoter. The putative transcripts and expressed proteins in the presence and absence of T7 promoter-mediated transcription are depicted below. To obtain T7 RNA polymerase and to demonstrate that plasmids can be recovered from stably transfected eukaryotic cell lines, we extracted plasmid DNA from BSR-T7/5 cells (https://web.expasy.org/cellosaurus/CVCL_RW96) using GeneJET Plasmid Miniprep kit (Thermo Scientific). E. coli (TOP10; Thermo Scientific) was used for amplification of the plasmid, and ZymoPURE II Plasmid Maxiprep kit (Zymo Research) for producing a maxiprep from a single colony. All steps were done according to the manufacturers’ guidelines. (B) To demonstrate that snake cells do not produce T7 promoter-driven transcripts, we transfected I/1Ki cells with the synthetic UGV-1 S segment (described above) with and without the T7 RNA polymerase plasmid isolated from BSR-T7/5 cells. We collected transfected cells at 1, 2, and 3 days posttransfection in RIPA buffer (50 mM Tris, 150 mM NaCl, 1% Triton X-100, 0.2% SDS, including Complete EDTA-free protease inhibitor cocktail [Roche]), quantified the protein concentration using BCA, and loaded 8 μg of protein per lane for SDS-PAGE separation and subsequent Western blotting. We probed the membrane with mouse anti-FLAG (left panel) and rabbit anti-HA (middle panel) and overlaid the signals (right panel). Anti-mouse AF680 and anti-rabbit IR800 served as secondary antibodies to enable recording the results with an Odyssey infrared imaging system. Download FIG S2, TIF file, 1.9 MB. Copyright © 2020 Szirovicza et al. 2020 Szirovicza et al. This content is distributed under the terms of the Creative Commons Attribution 4.0 International license.

### Replication of SDeV in human and snake cells.

**FIG 4:**
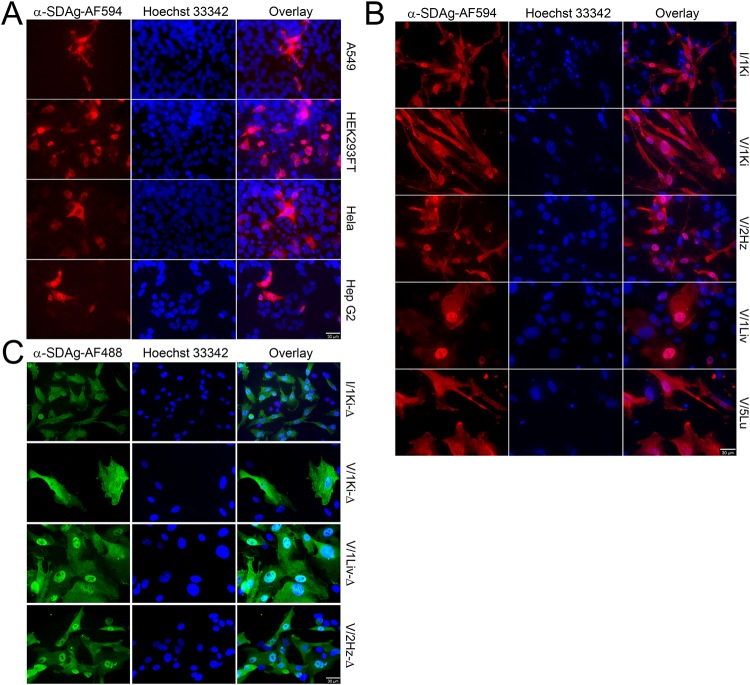
SDeV replicates in human and reptilian cell lines. (A) A549 (human lung carcinoma), HEK293FT (human embryonic kidney), HeLa (human cervical cancer), and HepG2 (human hepatocellular carcinoma) cells transfected with Δ-fwd (pCAGGS-SDeV-FWD) were stained at 5 days posttransfection for SDAg (anti-SDAg-AF594 [α-SDAg-AF594], left panels, red). Hoechst 33342 was used to visualize the nuclei. The panels on the right show an overlay of the three images. (B) Boid cell lines I/1Ki (kidney), V/1Ki (kidney), V/2Hz (heart), V/1Liv (liver), and V/5Lu (lung) transfected with Δ-fwd (pCAGGS-SDeV-FWD) were stained at 5 days posttransfection for SDAg (α-SDAg-AF594, left panels, red), and Hoechst 33342 was used to visualize the nuclei. The panels on the right show an overlay of the three images. (C) The transfected boid cells from panel B were allowed to grow, passaged three times, and stained for SDAg (α-SDAg-AF488, left panels, green), and Hoechst 33342 was used to visualize the nuclei. The panels on the right show an overlay of the two images. All images were taken at ×400 magnification using a Zeiss Axioplan 2 microscope, and a 30-μm bar is shown in the bottom right corner of each panel.

After demonstrating that replication of SDeV can be initiated in both boid and monkey kidney cells by transfecting the pCAGGS-SDeV-FWD plasmid, we wanted to test whether SDeV replication would also occur in human cell lines. We transfected human lung carcinoma (A549), hepatocellular carcinoma (HepG2), cervical cancer (HeLa), and embryonic kidney (HEK293FT) cell lines with the pCAGGS-SDeV-FWD plasmid and used IF to demonstrate the presence of SDAg. IF staining at 5 dpt showed cytoplasmic expression of SDAg in all cell lines studied (Fig. 4A). In addition, we found prominent SDAg staining in the nuclei of A549 and HepG2 cells. To study whether similar differences in SDAg localization would occur in snake cells, we transfected boid kidney (I/1Ki and V/1Ki), heart (V/2Hz), liver (V/1Liv), and lung (V/5Lu) cell lines with the pCAGGS-SDeV-FWD plasmid. We performed IF staining at 5 dpt and observed a variable SDAg expression pattern depending on the cell line (Fig. 4B). However, in all cell lines, SDAg was found in both the cytoplasm and nucleus. Curiously, the localization appeared to be more pronounced in the nucleus in liver and heart cell lines. With the current set of experiments, we cannot entirely rule out the possibility that the observed SDAg expression in mammalian cells is a consequence of leaky transcription from the T7 promoter, since some mammalian cells can use the T7 promoter for transcription (36, 37).

### Transfection of pCAGGS-SDeV-FWD into boid cells results in persistent SDeV infection.

After demonstrating that transfection of cultured snake cells with pCAGGS-SDeV-FWD induces SDeV replication, we wanted to study the effect of prolonged maintenance of the transfected cells under normal culture conditions. The extent of the cytopathic effect induced by the initial transfection varied considerably between the cell lines used, but we allowed the surviving cells to reach confluency. We passaged the cells until they reached a surface area of approximately 150 to 175 cm^2^, and all—except the lung cell line (V/5Lu)—revived within a few weeks after the transfection. IF to detect SDAg (Fig. 4C) showed its expression in all cell lines, but the number of SDeV-infected cells appeared to vary between the cell lines. For I/1Ki, V/1Liv, and V/2Hz cells, most cells displayed SDAg, indicating active replication, whereas for V/1Ki cells, only 5 to 10% of the cells were SDAg positive. The localization of SDAg varied between the different cell lines, but most often SDAg was found in both cytoplasm and nucleus, which is similar to what we observed *in vivo* (6). We named the persistently SDeV-infected cell lines I/1Ki-Δ, V/1Ki-Δ, V/1Liv-Δ, V/2Hz-Δ, and V/5Lu-Δ. Analysis of the cells by IF at approximately 6 months after the initial transfection showed that most cells express SDAg. To confirm that the observed SDAg expression is indeed due to permanent infection as opposed to transcription from the T7 promoter, we analyzed the cells for the presence of plasmid DNA. To demonstrate that the plasmid DNA would be detected by the chosen approach, we analyzed freshly transfected I/1Ki cells at 1, 4, and 7 dpt. As a further indication of gradual disappearance of the plasmid, we used quantitative PCR (qPCR) to demonstrate that the amount of plasmid DNA reduces proportionally when passaging the transfected cells. The results show that the generated cell lines do not possess detectable amounts of pCAGGS-SDeV-FWD (or -REV) plasmid (Fig. S3), verifying that the SDAg expression is indeed due to SDeV replication.

10.1128/mBio.03250-19.3 FIG S3 Permanently infected cell lines do not contain the original plasmid. (A) To demonstrate that SDAg expression is due to SDeV replication, we extracted plasmid DNA from I/1Ki, V/2Hz, V/1Liv, V/1Ki, I/1Ki-Δ, V/2Hz-Δ, V/1Liv-Δ, and V/1Ki-Δ cell lines, I/1Ki cells transfected with pCAGGS-SDeV-FWD (1, 4, and 7 days posttransfection), and brain homogenate-inoculated I/1Ki cells using GeneJET Plasmid Miniprep kit (Thermo Scientific). We analyzed the isolated DNA by PCR (primers, 5′-AT GCA GTA CGG CTG AAA GG-3′ and 5′-CCC ATA TGT CCT TCC GAG TG-3′) targeting a 337-bp region comprising of plasmid and insert and analyzed the PCR products on 2% agarose gel. The result shows detectable amount of plasmid DNA only in the freshly transfected I/1Ki cells. (B) To show that plasmid DNA is not amplified in the transfected cells, we passaged the pCAGGS-SDeV-FWD-transfected I/1Ki cells at different ratios (1/2, 1/3, 1/4, 1/6, 1/8, and 1/10), allowed them to reach confluency, detached the cells, quantified the cell suspension using TC20 cell counter (Bio-Rad), and used 10^6^ cells for plasmid DNA extraction. Maxima SYBR Green qPCR Master Mix (Thermo Scientific) with primers 5′-CAG CCA TTG CCT TTT ATG GT-3′ and 5′-TAC GGA TCT TCT CGC CAA CT -3′ served for quantification of the plasmid DNA. The bar graph shows that the plasmid DNA amount correlates with the passaging ratio, suggesting that after sequential passaging, the cell population would lose the plasmid DNA. (C) To demonstrate that the amount of SDAg does not depend on the amount of plasmid DNA, we analyzed the cells from the same set of samples by Western blotting. The membrane probed with anti-SDAg and pan-actin (a loading control) shows low variation in SDAg level. (D) Anti-SDAg IF staining of the above-described sample set shows low variation in the number of SDAg-expressing cells. Download FIG S3, TIF file, 2.4 MB. Copyright © 2020 Szirovicza et al. 2020 Szirovicza et al. This content is distributed under the terms of the Creative Commons Attribution 4.0 International license.

Encouraged by the findings that SDeV can establish persistent infection in snake cells, we tried the same approach for mammalian cells (Vero E6), but IF screening showed that this cell line was not able to maintain SDeV infection when cultured at 37°C. To determine whether temperature is an influencing factor, we kept the transfected Vero E6 cells at 30°C, but this had little or no effect on virus replication as judged by the number of cells displaying SDAg.

### Superinfection of I/1Ki-Δ cells with reptarenaviruses and hartmaniviruses produces infectious SDeV particles.

**FIG 5:**
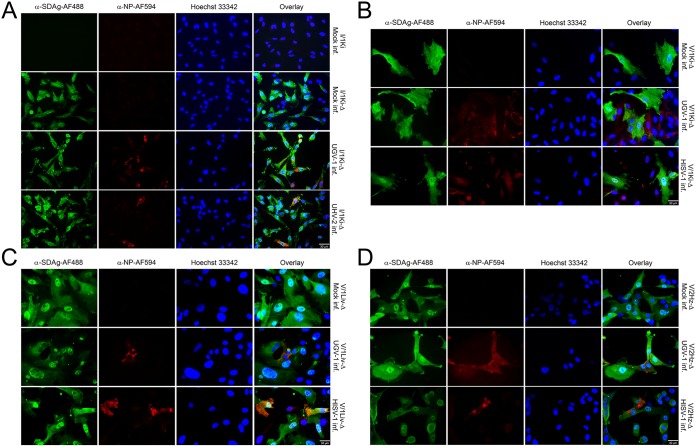
SDeV-infected cells can be superinfected with reptarenaviruses (UHV-2 and UGV-1) and hartmanivirus (HISV-1). (A) Mock-infected I/1Ki cells (boa kidney) and mock-, UGV-1-, and UHV-2-infected I/1Ki-Δ cells were stained for SDAg (anti-SDAg-AF488 [α-SDAg-AF488], left panels, green) and reptarenavirus NP (α-NP-AF594, second column, red). Hoechst 33342 was used to visualize the nuclei. The panels on the right show an overlay of the three images. (B) Mock-, UGV-1-, and HISV-1-infected V/1Ki-Δ cells (boa kidney) were stained for SDAg (α-SDAg-AF488, left panels, green), reptarenavirus NP (α-NP-AF594, second column, except bottom, red), or hartmanivirus NP (anti-HISV NP [1:3,000] and Alexa Fluor 594-labeled donkey anti-rabbit immunoglobulin [1:1,000], second column bottom panel, red). Hoechst 33342 was used to visualize the nuclei. The panels on the right show an overlay of the three images. (C) Mock-, UGV-1-, and HISV-1-infected V/1Liv-Δ cells (boa liver) were stained for SDAg (α-SDAg-AF488, left panels, green), reptarenavirus NP (α-NP-AF594, second column, except bottom, red), or hartmanivirus NP (anti-HISV NP [1:3,000] and Alexa Fluor 594-labeled donkey anti-rabbit immunoglobulin [1:1,000], second column bottom panel, red). Hoechst 33342 was used to visualize the nuclei. The panels on the right show an overlay of the three images. (D) Mock-, UGV-1-, and HISV-1-infected V/2Hz-Δ cells (boa heart) were stained for SDAg (α-SDAg-AF488, left panels, green), reptarenavirus NP (α-NP-AF594, second column, except bottom, red), or hartmanivirus NP (anti-HISV NP [1:3,000] and Alexa Fluor 594-labeled donkey anti-rabbit immunoglobulin [1:1,000], second column bottom panel, red). Hoechst 33342 was used to visualize the nuclei. The panels on the right show an overlay of the three images. All images were taken at ×400 magnification using a Zeiss Axioplan 2 microscope, and a 30-μm bar is shown in the bottom right corner of each panel.

As we had succeeded in isolating SDeV from the brain of a *B. constrictor* that showed no traces of a coinfecting hepadnavirus but instead carried several reptarenavirus and hartmanivirus large (L) and small (S) segments (6, 33), we hypothesized that SDeV would use arenaviruses as helpers for the formation of infectious particles. To demonstrate that the permanently SDeV-infected cell lines can be superinfected with reptarenaviruses and/or hartmaniviruses, we incubated I/1Ki-Δ cells with reptarenaviruses (University of Helsinki virus-2 [UHV-2] and University of Giessen virus-1 [UGV-1]) or a hartmanivirus (Haartman Institute Snake virus-1 [HISV-1]) and analyzed the cells by IF. Figure 5A shows that I/1Ki-Δ cells can indeed be superinfected with reptarenaviruses (UHV-2 and UGV-1). The localization of HDAg changes from nuclear to cytoplasmic during the viral life cycle (39), and we found that most I/1Ki-Δ cells display SDAg in the cytoplasm. However, some cells showed a granular nuclear SDAg staining, similarly to HDAg in human hepatocytes (40), and curiously, granules appeared less abundant in the reptarenavirus superinfected I/1Ki-Δ cells (Fig. 5A). Next, we tested whether the other permanently deltavirus-infected cell lines could be superinfected with reptarenavirus (UGV-1) or hartmanivirus (HISV-1). IF staining shows that we could indeed superinfect all permanently SDeV-infected cell lines (V/1Ki-Δ, V/1Liv-Δ, and V/2Hz-Δ) with both viruses (Fig. 5B to D). The shift of SDAg from the nucleus to the cytoplasm as a result of superinfection, suggested with experiments done on I/1Ki-Δ cells, appeared less clear in the other cell lines tested, and further studies are needed to determine whether coinfection indeed affects the localization of SDAg.

**FIG 6:**
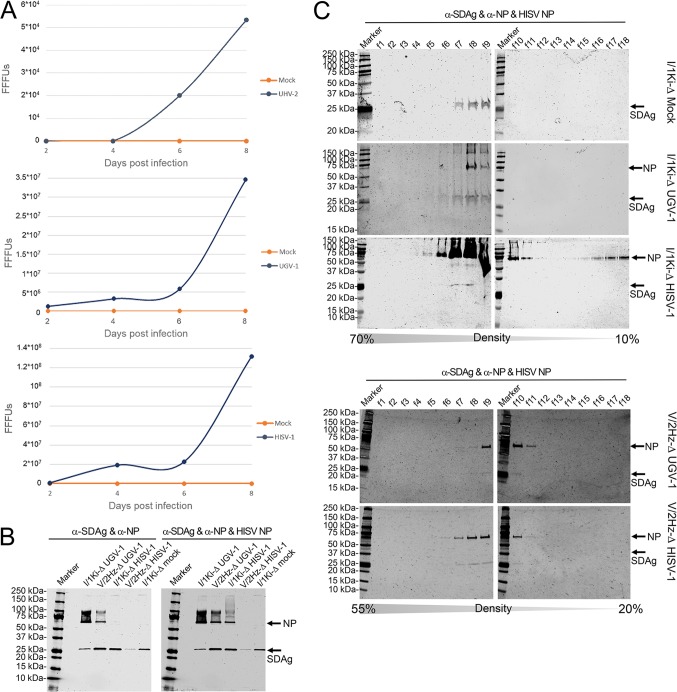
Superinfection of permanently SDeV-infected boid cells (I/1Ki-Δ and V/2Hz-Δ) induced production of infectious SDeV particles. (A) Supernatant collected from mock-, UHV-2 (top), UGV-1 (middle), and HISV-1 (bottom) superinfected I/1Ki-Δ cells at 2, 4, 6, and 8 days postinfection (dpi) were titrated on clean I/1Ki cells. The *y* axis shows the number of fluorescent focus-forming units (FFFUs) per milliliter of culture medium. (B) Supernatants collected from mock-, UHV-2, UGV-1, and HISV-1 superinfected I/1Ki-Δ cells and UGV-1 and HISV-1 superinfected V/2Hz-Δ cells were pelleted by ultracentrifugation and analyzed by Western blotting. The left panel shows anti-SDAg staining, and the right panel shows anti-SDAg, anti-reptarenavirus NP, and anti-hartmanivirus NP staining. (C) Pelleted supernatants collected from mock-, UGV-1, and HISV-1 superinfected I/1Ki-Δ cells and UGV-1 and HISV-1 superinfected V/2Hz-Δ cells were subjected to density gradient ultracentrifugation, and the fractions collected from the bottom of the tubes were analyzed by Western blotting using anti-SDAg and anti-reptarenavirus NP staining (for mock and UGV-1) or anti-SDAg and anti-hartmanivirus NP staining (for HISV-1). The arrows indicate the locations of SDAg and reptarenavirus or hartmanivirus NP. Precision Plus Protein Dual Color Standards (Bio-Rad) served as the markers for both panels B and C, and the results were recorded using the Odyssey infrared imaging system (Li-Cor).

Next, we evaluated whether reptarena- or hartmanivirus superinfection of I/1Ki-Δ cells can induce the formation of infectious SDeV particles. We chose to use I/Ki-Δ cells for the experiment, since we have shown that this cell line is permissive for several viruses (30–33). We inoculated I/1Ki-Δ cells with UHV-2, UGV-1, or HISV-1, collected supernatant up to 8 dpi, and analyzed it for infectious particles. Subsequently, we inoculated a fresh monolayer of clean I/1Ki cells with the supernatants and used supernatant collected from nonsuperinfected (mock) I/1Ki-Δ cells as the control. At 2 to 5 dpi, we IF stained the cells for SDAg and counted the number of fluorescent foci at each time point; examples of IF staining are shown in Fig. S4. The nonsuperinfected I/1Ki-Δ cells did not produce infectious particles, while the cells superinfected with either reptarenaviruses or hartmanivirus produced infectious SDeV particles (Fig. 6A). The production of infectious SDeV particles appeared to be the most efficient in HISV-1-infected cells, while UHV-2-infected cells produced the smallest number of infectious SDeV particles (Fig. 6A). The observed difference between the numbers of infectious SDeV particles produced in UHV-2- versus UGV-1-infected cells might be related to the comparatively lower replication rate of UHV-2, as reported in our previous study (33).

10.1128/mBio.03250-19.4 FIG S4 SDAg expression overview of I/1Ki cells inoculated with supernatants collected from arenavirus superinfected or glycoprotein (GP) transfected I/1Ki-Δ cells. To demonstrate that arenavirus superinfection and/or GP transfection of I/1Ki-Δ cells induces production of infectious SDeV particles, we stained I/1Ki cells inoculated with afore mentioned supernatants for the presence of SDAg. We also wanted to provide an overview of the SDAg staining in the inoculated cells, to rule out the possibility that the staining would be due to antigen carryover from the permanently SDeV infected cells. (A) An overview of the plate, from which the titers of infectious SDeV particle formation following GP transfections were obtained. Only the green channel (anti-SDAg) is shown for clarity. (B) Close-ups from selected wells (indicated by blue frames in panel A) demonstrating that the positive cells show similar staining to that of permanently SDeV infected cells (compare to, e.g., Fig. 1, Fig. 4, or Fig. 5). (C) Close-ups to demonstrate that I/1Ki cells infected with supernatants collected from arenavirus (reptarenavirus UGV-1 and hartmanivirus HISV-1) superinfected I/1Ki-Δ cells produces similar SDAg staining pattern. All images were acquired from 96-well plates (ViewPlate-96 F TC; PerkinElmer) using Opera Phenix High Content Screening System (PerkinElmer) with 20× water immersion objective. Download FIG S4, TIF file, 2.5 MB. Copyright © 2020 Szirovicza et al. 2020 Szirovicza et al. This content is distributed under the terms of the Creative Commons Attribution 4.0 International license.

In an attempt to isolate and demonstrate SDeV particles, we subjected the cell culture supernatants collected from mock, UHV-2, UGV-1, and HISV-1 superinfected I/1Ki-Δ and UGV-1 and HISV-1 superinfected V/2Hz-Δ cells to ultracentrifugation and used WB to demonstrate SDAg in the pellets obtained. The results show that SDAg was pelleted not only from the supernatants of reptarenavirus and hartmanivirus superinfected I/1Ki-Δ and V/2Hz-Δ cells but also from the mock superinfected I/1Ki-Δ cells (Fig. 6B). However, as demonstrated by the inoculation experiments (Fig. 6A), the particles secreted from mock superinfected cells were noninfectious. As an attempt to separate the SDeV particles from the superinfecting reptarenavirus or hartmanivirus particles, we subjected the pelleted material to density gradient ultracentrifugation. However, even with different sucrose gradients and centrifugation times (10 to 70% sucrose gradient for 18 h and 4 h and 20 to 55% sucrose gradient for 1.5 h), we were unable to separate SDeV and reptarenaviruses or hartmaniviruses into different fractions (Fig. 6C).

**FIG 7:**
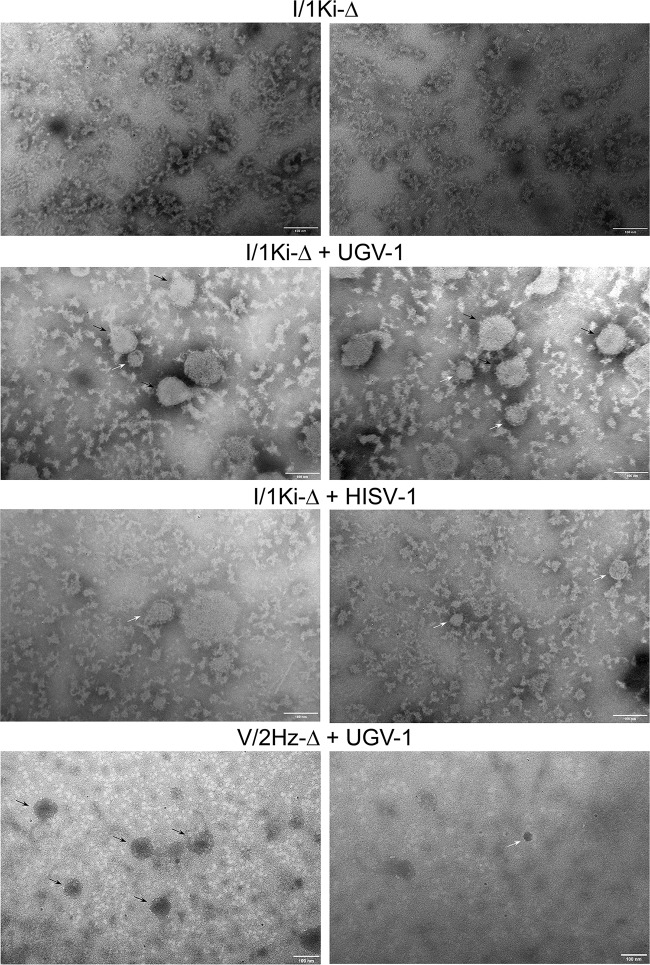
Transmission electron microscopy (TEM) of pelleted supernatants. Persistently SDeV-infected I/1Ki-Δ cells were inoculated with medium collected from clean I/1Ki cells (mock) or superinfected with UGV-1 or HISV-1. V/2Hz-Δ cells were superinfected either with UGV-1 or HISV-1. The cell culture medium was collected at 2- to 3-day intervals until 14 days postinfection, after which the supernatants were pooled and filtered, followed by ultracentrifugation to pellet the virus particles. After resuspending the pelleted material, an aliquot of the pelleted material was prepared for TEM with negative staining. The top panels show the material pelleted from mock-infected I/1Ki-Δ cells, the second row of panels show the material pelleted from UGV-1-infected I/1Ki-Δ cells, the third row of panels show the material pelleted from HISV-1-infected I/1Ki-Δ cells, and the bottom panels show the material pelleted from UGV-1-infected V/2Hz-Δ cells. The black arrows in the figure panels point to UGV-1 particles, and the white arrows show putative SDeV particles as judged by size. The images were taken by using a JEOL JEM-1400 transmission electron microscope at ×200,000 magnification.

Because separation of SDeV and arenavirus particles was unsuccessful, we studied the material pelleted from mock, UGV-1 or HISV-1 superinfected I/1Ki-Δ and UGV-1 or HISV-1 superinfected V/2Hz-Δ cell culture supernatants for the presence of secreted particles by electron microscopy (EM). Examination of negatively stained samples at ×200,000 magnification showed particles in the pellets obtained from the supernatants of UGV-1 and HISV-1 superinfected I/1Ki-Δ cells and in those obtained from UGV-1 superinfected V/2Hz-Δ cells. The pellets from mock-superinfected I/1Ki-Δ and HISV-1 superinfected V/2Hz-Δ cell culture supernatants were devoid of particles. The pellets obtained from UGV-1 superinfected I/1Ki-Δ and V/2Hz-Δ cells showed the presence of smaller (30- to 60-nm) and larger (∼100-nm) particles, which was also the case for the pellets obtained from HISV-1 superinfected I/1Ki-Δ cell culture supernatants (Fig. 7). The size of arenavirus particles is highly variable, ranging from 40 to 200 nm in diameter (41); however, we speculate that the smaller particles with diameters in the range of 30 to 60 nm could represent SDeV particles.

### Transfection of I/1Ki-Δ cells with viral glycoproteins induces production of infectious particles.

**FIG 8:**
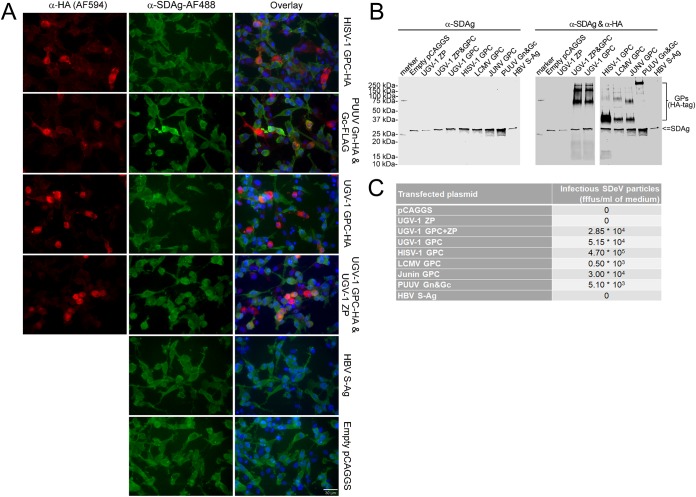
Infectious SDeV particles are formed when I/1Ki-Δ cells are transfected with viral glycoproteins. (A) I/1Ki-Δ cells transfected with HISV-1 GPC (top row), Puumala virus glycoproteins (PUUV Gn&Gc, second row), UGV-1 GPC (third row), UGV-1 ZP and GPC (fourth row), HBV S-Ag (fifth row), and empty pCAGGS-MCS plasmid (bottom row) were stained for HA tag (anti-HA [1:4,000] and Alexa Fluor 594-labeled donkey anti-mouse immunoglobulin [1:1,000], left panels, red) and SDAg (α-SDAg-AF488, middle panels, green). Hoechst 33342 was used to visualize the nuclei. The panels on the right show an overlay of the two images. A 30-μm bar is shown in the bottom right corner. All images were taken at ×400 magnification using a Zeiss Axioplan 2 microscope. (B) Supernatants collected from I/1Ki-Δ cells transfected with empty pCAGGS-MCS plasmid, UGV-1 ZP, UGV-1 GPC and ZP, UGV-1 GPC, HISV-1 GPC, LCMV GPC, JUNV GPC, PUUV glycoproteins, and HBV S-Ag were pelleted by ultracentrifugation and analyzed by Western blotting. The left panel shows anti-SDAg staining, and the right panel shows anti-SDAg and anti-HA staining. (C) Supernatants collected from I/1Ki-Δ cells transfected with empty pCAGGS-MCS plasmid, UGV-1 ZP, UGV-1 GPC and ZP, UGV-1 GPC, HISV-1 GPC, LCMV GPC, JUNV GPC, PUUV glycoproteins, and HBV S-Ag were titrated on clean I/1Ki cells. The plasmid used for transfection is shown in the left column, and the corresponding SDeV titer (in fluorescent focus-forming units [fffus] per milliliter) is shown in the right column.

Because superinfection of I/1Ki-Δ cells with both reptarenaviruses and hartmaniviruses resulted in the production of infectious SDeV particles, we wanted to study which of the structural proteins are required for the particle formation. While the envelope of both classical arenaviruses (genus *Mammarenavirus*) and reptarenaviruses comprises both matrix protein (ZP) and spike complexes, the envelope of hartmaniviruses lacks the ZP (33). Glycoproteins GP1 and GP2, encoded as a GPC, form the major portion of the spike complex, which in the case of mammarenaviruses and presumably, hartmaniviruses comprises also a stable signal peptide (33). We started by transfecting I/1Ki-Δ cells with the GPCs of HISV-1, Puumala virus (PUUV, an orthohantavirus), and UGV-1 (with and without cotransfected ZP). Additionally, we transfected the cells with HBV S-antigen (S-Ag)-bearing plasmid. We included PUUV GPC, since orthohantaviruses, like mammarenaviruses, are known to induce persistent infection in their rodent hosts and could thus represent a potential helper virus. Additionally, the GPC of orthohantaviruses is similar to that of arenaviruses in the sense that it gives rise to two glycoproteins, Gn and Gc, which form the spike complex (42). We could demonstrate the expression of glycoproteins using IF staining for all glycoproteins except HBV S-Ag (Fig. 8A), for which we did not include the HA epitope tag, and staining with serum from a vaccinated person produced extensive background staining. We found SDAg predominantly in the cytoplasm; however, many of the nontransfected cells displayed a punctate SDAg reaction in the nucleus. We could not conclude whether the expression of viral glycoproteins affects the localization of SDAg.

Moreover, we transfected I/1Ki-Δ cells with empty pCAGGS plasmid, UGV-1 ZP, UGV-1 GPC and ZP, UGV-1 GPC, HISV-1 GPC, lymphocytic choriomeningitis virus (LCMV) GPC, Junin virus (JUNV) GPC, PUUV glycoproteins, and HBV S-Ag and collected the supernatant for up to 7 dpt. We used ultracentrifugation to pellet the material secreted from the transfected cells and analyzed the pellets by WB. Transfection of the cells with glycoproteins appeared to enhance the secretion of SDAg; furthermore, by employing the HA tag, we could demonstrate the expressed viral glycoproteins in the pelleted material by WB (Fig. 8B). To study whether expression of glycoproteins had induced formation of infectious SDeV particles, we infected naïve I/1Ki cells with the collected supernatants. The results show that the cells transfected with an empty plasmid, UGV-1 ZP, or HBV S-Ag did not produce infectious particles (Fig. 8C). In contrast, the cells cotransfected with UGV-1 ZP and GPC did produce infectious particles. Notably, expression of UGV-1 GPC alone produced larger amounts of infectious particles, indicating that the expression of ZP is not needed for the production of infectious SDeV particles (Fig. 8C). Similar to the superinfection experiments, in which HISV-1 was found as the most effective helper virus, the expression of HISV-1 GPC induced the highest concentration of infectious SDeV particles. We could also further show that the expression of the mammarenavirus and even orthohantavirus GPCs induced the production of infectious particles. We assume that the infectious titer of SDeV particles carrying LCMV, JUNV, or PUUV glycoproteins might actually be higher, since these viruses might not enter I/1Ki cells as efficiently as the snake-derived viruses (UGV-1 and HISV-1).

## DISCUSSION

Until 2018, the genus *Deltavirus* was represented by a single species, HDV, which was intimately linked with HBV infection. HDV is a satellite virus of HBV, which mostly targets the human liver (25); hence, HDV infection is mostly associated with liver disease. HDV infection affects around 20 million people worldwide (43); however, it is currently a somewhat neglected disease due to the fact that HBV vaccination is thought to also provide protection against HDV infection. The recent discovery of deltaviruses in the absence of hepadnaviral coinfection across a wide range of taxa (5, 6, 8) provided the first indications that deltaviruses could be much more enigmatic than originally thought. We found SDeV in several tissues of the infected snakes, indicating that the virus has a broad tissue tropism and further implying that SDeV does not rely on a hepadnavirus as its helper (6). Indeed, in 2019, Perez-Vargas and colleagues demonstrated that HDV can form infectious particles utilizing the envelope glycoproteins from various viral species (28). We originally found SDeV in coinfection with reptarena- and hartmaniviruses (6), and wanted to study whether these viruses could provide the glycoprotein-decorated envelope for the production of infectious SDeV particles.

Here, we generated plasmid constructs bearing the SDeV genome in either the genomic or antigenomic orientation and could demonstrate that transfecting these plasmids into cultured snake, monkey, or human cells initiates SDeV replication. These findings imply that SDeV replication itself is not limited to any particular cell type but instead would be restricted by the envelope borrowed from the co-/superinfecting helper virus. Similarly, HDV infection is likely not restricted to the liver since Perez-Vargas and colleagues demonstrated infectious HDV particle formation in coinfection with various enveloped viruses (28). By passaging of the cells transfected with SDeV constructs, we could demonstrate that, at least in cell culture, deltaviruses can rather easily establish a persistent infection. The persistently infected cell lines allowed us to imitate SDeV infection *in vitro* and to overcome the problems faced in human hepatitis virus research, i.e., the lack of a solid cell culture system that allows viral infection and propagation (44). Moreover, our results not only show that SDeV can establish and maintain a persistent infection *in vitro* but also indicate that helper virus is not required for persistent infection. HBV-independent persistence of HDV and subsequent rescue by HBV superinfection has been shown in woodchucks (45), chimpanzees (46), and mice (47, 48). Also, HDV can persist in human hepatocytes (in the livers of humanized mice) without its helper virus and potentially be rescued by a later HBV infection (48). The persistence of SDeV in snake cell lines shown in this study and the persistence of HDV in human hepatocytes (49) resemble each other in the sense that the accumulation of positive cells appears to rely on cell division rather than a helper virus.

The persistently SDeV-infected cell lines enabled us to study the release of SDAg in boa constrictor cells superinfected with reptarenaviruses and hartmanivirus. To our surprise, we found I/1Ki-Δ cells to secrete SDAg even without superinfection. However, cells could not be infected using the material released from nonsuperinfected cells. We also observed that the cellular distribution of SDAg differed between cell lines, ranging from mostly nuclear in liver and heart (V/1Liv and V/2Hz) to mainly cytoplasmic in lung and kidney (V/5Lu and V/1Ki) boa constrictor cell lines. The cellular distribution of HDAg changes during the viral life cycle, as the viral ribonucleoproteins are transported back and forth between the nucleus and the cytoplasm; however, this transport is influenced by different factors (39). It could be that some cell lines express proteins capable of triggering redistribution of SDAg. Such proteins could include, e.g., hepadnaviral and lentiviral glycoproteins integrated into the host’s genome. In fact, infectious HDV particles can be formed via low-level expression of genome-integrated HBV S-Ag (50). It remains unclear to what degree endogenous viral elements found in animals, plants, and fungi could also contribute to the formation of infectious deltavirus particles and thereby also to their spread. Alternatively, it could be speculated that the observed cellular distribution of SDAg in kidney and lung cells would be due to secretion within vesicles. In support of the latter hypothesis, we observed that the SDAg secreted from I/1Ki-Δ cells migrates to the same fractions as reptarenaviruses and hartmaniviruses in density gradient ultracentrifugation. By superinfecting I/1Ki-Δ cells with reptarenaviruses and hartmanivirus, we could induce production of infectious SDeV particles. We could further demonstrate that the expression of glycoproteins alone induces formation of infectious SDeV particles, even though the ZP of arenaviruses is known to contribute to the budding of virions (51). The cotransfection of ZPs did not improve but instead reduced the efficiency of SDeV particle production, which could in theory be explained by the ability of ZPs to promote budding and formation of particles without viral glycoproteins. Interestingly, hartmanivirus infection or the expression of hartmanivirus glycoprotein seemed to induce the most efficient production of infectious SDeV particles. The dissimilarity could be due to the suggested differences between reptarenavirus (no cytoplasmic tail) and hartmanivirus (cytoplasmic tail with putative late domains) glycoproteins (33). We could further show that the expression of mammarenavirus (LCMV or JUNV) and hantavirus (PUUV) glycoproteins also induces formation of infectious SDeV particles. We have also applied the same approach for HDV and observed that the expression of arenavirus and hantavirus glycoproteins is sufficient for the production of infectious particles. Since parallel findings using hepacivirus, flavivirus, and vesiculovirus helpers have recently been published by Perez-Vargas and coauthors (28), we have decided not to include our results regarding HDV in this article. However, taken together, these findings imply that various deltaviruses would rely on several different helper viruses to complete their life cycle.

These newly described characteristics of deltaviruses raise numerous questions regarding the range of possible helper viruses, and factors contributing to deltavirus-glycoprotein interactions and subsequent infectious particle formation. Further studies will need to address which viruses can act as deltavirus helpers. It is tempting to speculate that deltaviruses would be opportunistic microbes, the exit (or infectious particle formation) of which would rely on persistent, latent, or recurring infections by enveloped viruses. Such infections could include arenaviruses and hantaviruses, both of which cause persistent infection in rodents (52, 53). In humans, examples of persistent viral infections could include HBV, hepatitis C virus, and human immunodeficiency virus, the first two of which have already been demonstrated to induce the formation of infectious HDV particles (28). Additionally, recurring infections such as those caused by ortho-, paramyxo-, or coronaviruses or latent infections caused by, e.g., herpesviruses, could contribute to the spread of deltaviruses. Thus, it seems fair to speculate that HDV could present merely the tip of an iceberg in terms of human deltavirus infections. This theory would be supported by the detection of HDAg in the absence of HBV from the salivary glands of Sjögren’s syndrome patients (54). As the number and variety of deltaviruses are greater than earlier assumed, it would be worth to explore whether deltaviruses are underlying or exacerbating agents in both animal and human diseases. It is tempting to speculate that a common ancestor of deltaviruses could be found among viroids of higher plants, as they show the highest similarities with deltaviruses (4). Since a deltavirus has already been identified in termites (8), one could hypothesize that the deltavirus ancestor was transmitted to Animalia from plants. Indeed, Van Bogaert and colleagues showed the presence of viroids in aphids feeding on infected plants (55), which could support the above hypothesis. However, the questions regarding the origin of deltaviruses will need to be addressed by further studies.

Deltaviruses are likely widespread both worldwide and across different taxa. So far, novel deltaviruses have been found in snakes, birds, fish, amphibians, and invertebrates (5, 6, 8). These findings add to the previously limited knowledge about the origin and evolution of HDV (7). Also, the findings on the infidelity of HDV to HBV (28) indicate that many new doors have lately been opened in the field of deltavirus research.

## MATERIALS AND METHODS

### Cell lines and viruses.

We used the following established cell lines: human hepatocellular carcinoma, HepG2 (American Type Culture Collection [ATCC]); African green monkey kidney, Vero E6 (ATCC); human embryonic kidney, HEK293FT (ThermoFisher Scientific); human lung carcinoma, A549 (ATCC). The *Boa constrictor* kidney cell line I/1Ki was described by Hetzel et al. (30) in 2013. Additionally, we established the following cell lines by applying techniques described by Hetzel et al. (30); *B. constrictor* kidney, V/1Ki; *B. constrictor* liver, V/1Liv; *B. constrictor* lung, V/5Lu; and *B. constrictor* heart, V/2Hz.

To maintain the cultured mammalian and I/1Ki cell lines, we used Eagle minimal essential medium (MEM) supplemented with 10% fetal bovine serum, 200 mM l-glutamine, 100 μg/ml of streptomycin, and 100 U/ml of penicillin, while for the other snake cell lines, we used Dulbecco’s modified Eagle medium (DMEM) with high glucose supplemented with 15% fetal bovine serum, 200 nM l-alanyl l-glutamine, 100 μg/ml of streptomycin, and 100 U/ml of penicillin. We kept the cells in incubators with 5% CO_2_ and at 30°C or 37°C.

To obtain cell lines persistently infected with SDeV, we passaged the cells (culturing conditions as described above) transfected with plasmid bearing two copies of the SDeV genome (described below) until we obtained a confluent 175-cm^2^ flask and were able to prepare ampoules for storage. The following permanently infected cell lines were generated: I/1Ki-Δ, V/1Ki-Δ, V/1Liv-Δ, V/2Hz-Δ, and V/5Lu-Δ.

For superinfection studies, we used two reptarenaviruses, University of Helsinki virus-2 (UHV-2) (33) and University of Giessen virus-1 (UGV-1) (31), and one hartmanivirus, Haartman Institute Snake virus-1 (HISV-1) (33).

### Cloning, plasmids, and recombinant protein expression.

We ordered a synthetic gene from Gene Universal bearing the SDeV genome (GenBank accession no. MH988742.1) in duplicate (starting from residue 216 and ending at 215, i.e., exactly two copies of the genome), with the T7 promoter (5′-TAATACGACTCACTATAGG-3′) after the SDeV genome, and EcoRV restriction sites at both ends. We followed the manufacturer’s protocols throughout cloning. We used FastDigest EcoRV (ThermoFisher Scientific), agarose gel electrophoresis, and the GeneJET Gel extraction kit (ThermoFisher Scientific) to purify the synthetic insert. To subclone the synthetic insert to pCAGGS/MCS as described by Niwa et al. (56), we used FastDigest EcoRI and XhoI (both ThermoFisher Scientific) to linearize the plasmid, T4 DNA ligase (ThermoFisher Scientific) to blunt the 5′ and 3′ overhangs, and the GeneJET Gel extraction kit (ThermoFisher Scientific) to purify the plasmid after agarose gel electrophoresis. We ligated the insert to pCAGGS/MCS using T4 DNA ligase (ThermoFisher Scientific), transformed chemically competent Escherichia coli (DH5α strain) by standard methods, plated the bacteria on Luria broth agar plates with 100 μg/ml of ampicillin, picked single colonies after overnight (O/N) cultivation at 37°C into 5 ml of 2×YT medium (16 g/liter tryptone, 10 g/liter yeast extract, 5 g/liter NaCl), prepared minipreps from 2 ml of O/N cultivation at 37°C using the GeneJET plasmid miniprep kit (ThermoFisher Scientific), checked for the presence of insert using restriction digestion and agarose gel electrophoresis, Sanger sequenced (DNA Sequencing and Genomic Laboratory, Institute of Biotechnology, University of Helsinki) the plasmids to obtain clones with the insert in the genomic and antigenomic orientation, and used ZymoPURE II plasmid Maxiprep kit (Zymo Research) to obtain plasmid stocks for transfection.

For recombinant expression of HBV S-Ag, we ordered a synthetic gene (from GeneUniversal) covering the residues 2848 to 3215 and 1 to 835 of HBV (GenBank accession no. JX079936.1), which encode large, middle, and small antigen (S-Ag), with 5´ EcoRI and 3´ XhoI restriction sites, subcloned the insert into pCAGGS/MCS (56) and prepared plasmid stocks as described above. We also ordered codon-optimized (for human) synthetic genes (from GeneUniversal) based on UGV-1 ZP (NCBI protein accession no. AKN10693.1), with 5´ EcoRI and 3´ XhoI restriction sites and cloned the gene to pCAGGS/MCS for expression as described above. To clone UGV-1 GPC and HISV-1 GPC, we used reverse transcription-PCR (RT-PCR) amplification (reverse transcription with RevertAid reverse transcriptase [ThermoFisher Scientific]; PCR with Phusion Flash high-fidelity PCR master mix [ThermoFisher Scientific]) of RNA. RNA was extracted (by using GeneJET RNA extraction kit; ThermoFisher Scientific) with the following primers to generate inserts: UGV-GPC-fwd (5´-AAAGAATTCATGGCAGGTCACCTCAACCG-3´), UGV-GPC-rev (5´-TTTATGCATCCCCGTCTCACCCAGTTGC-3´), HISV-1 GPC-fwd (5´-AAAGAATTCATGGGGGCACTTGTGTCC-3´), and HISV-1 GPC-rev (5´-GGAGGTACCCCGTATTTTTCAATGGGACA-3´). The inserts were purified by using the GeneJET PCR purification kit (ThermoFisher Scientific), restriction digested the inserts with FastDigest EcoRI and SmaI (ThermoFisher Scientific) for UGV-1 GPC and FastDigest EcoRI and KpnI (ThermoFisher Scientific) for HISV-1 GPC. The inserts were purified as described above, ligated with Thermo Selective alkaline phosphatase-treated (ThermoFisher Scientific) pCAGGS-HA (57), and linearized with the respective restriction enzymes. The plasmid stocks were prepared as described above. Lymphocytic choriomeningitis virus (LCMV) and Junin virus (JUNV) GPCs were amplified using primers 5′-AATTCAATTGACCATGGGTCAGATTGTG-3′ and 5′-AATTCCCGGGGCGTCTTTTCCAGAC-3 (LCMV GPC) or 5′-AATTGAGCTCACCATGGGGCAGTTCATT-3′ and 5′-AATTCCCGGGGTGTCCTCTACGCCA-3′ (JUNV GPC) and cloned using EcoRI/MfeI and SmaI (LCMV GPC) or SacI and SmaI (JUNV GPC) into pCAGGS-HA. Plasmids were verified by sequencing. To clone Puumala virus (PUUV) orthohantavirus Gn (residues 1 to 658 of NCBI protein accession no. CCH22848.1) and Gc (residues 637 to 1148 of CCH22848.1), we used primers PUUV-Gn-fwd (5´-AATAGAATTCATGGGAAAGTCCAGCCCCGTGT-3´), PUUV-Gn-rev (5´-TCCCGGGTGCGCTGGCGGCCCACA-3´), PUUV-Gc-fwd (5´-AAGAGAATTCATGTTCTTCGTGGGCCT-3´), and PUUV-Gc-rev (5´-ATTCCCGGGCTTGTGCTCCTTC-3´) to PCR amplify (Phusion Flash high-fidelity PCR master mix; ThermoFisher Scientific) the inserts from codon-optimized PUUV GPC described by Iheozor-Ejiofor et al. (58), ligated the inserts employing EcoRI and SmaI restriction sites to both pCAGGS-HA and pCAGGS-FLAG (59), and prepared plasmid stocks as described above.

### Affinity purification and labeling of antibodies.

To enable the simultaneous use of two rabbit antibodies in immunofluorescence (IF) staining, we used recombinant UHV-1 NP and snake delta antigen (SDAg) to generate affinity-purified IgG fractions for subsequent labeling with fluorescent dyes. For the affinity purification, we coupled baculovirus-expressed recombinant UHV NP (60) and E. coli-expressed SDAg (6) to CNBr-activated Sepharose 4 Fast Flow (GE Healthcare Life Sciences) following the manufacturer’s protocol. We used a protocol described by Korzyukov et al. (61) to affinity purify the antibodies. After dialysis and concentration of the antibodies, we labeled the affinity-purified fractions using Alexa Fluor 488 tetrafluorophenyl (TFP) ester (ThermoFisher Scientific) or Alexa Fluor 594 succinimidyl (NHS) ester (ThermoFisher Scientific) following the manufacturer’s recommendation. The labeled antibodies were purified by passing through disposable PD 10 desalting columns (GE Healthcare Life Sciences), concentrated using Amicon Ultra-15 centrifugal filter units (Millipore) with 50,000 (50K)-nominal-molecular-weight cutoff, mixed with glycerol (final concentration, 50% [vol/vol]), and kept at −20°C for short-term storage and at −80°C for long-term storage. To determine optimal dilutions, we titrated the antibodies on clean and infected I/1Ki-Δ cells (SDeV with and without reptarenavirus infection), the antibodies generated (dilution range in parentheses) are anti-NP-AF488 (1:200 to 1:400), anti-NP-AF594 (1:200 to 1:400), anti-SDAg-AF488 (1:400 to 1:800), and anti-SDAg-AF594 (1:400 to 1:800).

### SDS-PAGE and immunoblotting.

We performed sodium dodecyl sulfate-polyacrylamide gel electrophoresis (SDS-PAGE) (self-prepared gels and 4 to 20% Mini-PROTEAN TGX gels from Bio-Rad) and immunoblotting using methods described by Korzyukov et al. (61). The antibody dilutions used were 1:2,500 to 1:20,000 for the rabbit anti-SDAg polyclonal antibody (pAb) (6), 1:4,000 for the mouse anti-HA-tagged monoclonal antibody (Mab) (AE008; ABclonal), 1:10,000 for Alexa Fluor 680-labeled donkey anti-rabbit (IgG) and anti-mouse (IgG) (ThermoFisher Scientific), and 1:10,000 for IRDye 800CW donkey anti-rabbit (IgG) and anti-mouse (IgG) (Li-Cor Biosciences). For staining of β-actin as a loading control, we used Lab Vision pan-actin mouse monoclonal antibody (ThermoFisher Scientific) at 1:200 dilution. The Odyssey infrared imaging system (Li-Cor Biosciences) was used to record the results.

### Transfection of cultured cells.

For transfection of *B. constrictor* cell lines, we used Lipofectamine 2000, and we used Fugene HD (Promega) for mammalian cells. When using Lipofectamine 2000, we prepared the transfection mixes by diluting the plasmid stock in OptiMEM (ThermoFisher Scientific) to yield 500 ng/50 μl, mixed 2.75 to 3.0 μl of Lipofectamine 2000 in 47 μl of OptiMEM (ThermoFisher Scientific), combined the two mixtures by pipetting up and down, and allowed the complexes to form 15 to 30 min at room temperature (RT). When using Fugene HD, we prepared the plasmid solution as described above, added 1.75 μl of Fugene HD, mixed by pipetting, and allowed the complexes to form (15 to 30 min at RT). The above recipes were used for transfecting 2 cm^2^ of cells and scaled up based on the desired cell surface area. After preparing the transfection reagent-plasmid complexes, we detached 80 to 90% confluent cell layers using Gibco trypsin-EDTA (0.25%; ThermoFisher Scientific) and pelleted the cells by centrifugation (3 to 4 min at 500 × *g*). We resuspended the cells in fully conditioned cell culture medium (described above) to yield a cell density of approximately 2 cm^2^/ml (calculated from the original surface area, e.g., cells from a 75-cm^2^ bottle would be resuspended in 37.5 ml) and mixed 1 ml of cell suspension with the transfection mix by pipetting. We incubated the resulting cell suspension and transfection mixture for 15 to 30 min at RT, plated the cells, and replaced the medium after 6-h incubation.

### Immunofluorescence staining.

We used black 96-well plates (ViewPlate-96 F TC; PerkinElmer) or 13-mm coverslips to grow the cells for IF staining. For collagen coating, we incubated the coverslips or plates O/N at +4°C with 0.1 mg/ml of collagen I from rat tail (BD Biosciences) in 0.25% acetic acid. To fix the cells, we removed the culture medium, added 4% paraformaldehyde (PFA) in phosphate-buffered saline (PBS) (pH 7.4), incubated for 10 min at RT, washed once with Tris-buffered saline (TBS) (50 mM Tris, 150 mM NaCl [pH 7.4]), permeabilized and blocked (0.25% Triton X-100 [Sigma Aldrich], 3% bovine serum albumin [BSA] [ThermoFisher Scientific] in TBS) for 5 to 10 min at RT and washed once with TBS. For IF staining, we incubated the cells with the primary antibodies diluted in TBS with 0.5% BSA for 60 to 90 min at RT, washed three times with TBS, incubated 45 min with the secondary antibody diluted in TBS with 0.5% BSA, washed three times with TBS, once with Hoechst 33342 diluted in TBS, once with TBS, twice with Milli-Q water (Millipore), and mounted the coverslips with Prolong Gold Antifade Mountant (ThermoFisher Scientific) or added 50 μl/well of 90% glycerol, 25 mM Tris-HCl (pH 8.5) for the 96-well plates. For primary antibodies, we used the following dilutions: 1:7,500 for anti-SDAg (6), 1:2,500 for anti-NP (60), 1:2,500 for anti-HISV NP (33), 1:250 for monoclonal anti-HA (ABclonal). For secondary antibodies, we used the following dilutions: 1:1,000 for Alexa Fluor 488- or 594-labeled donkey anti-rabbit immunoglobulin (ThermoFisher Scientific) and 1:1,000 for Alexa Fluor 488- or 594-labeled donkey anti-mouse immunoglobulin (ThermoFisher Scientific).

### Virus purification.

We used ultracentrifugation to pellet viruses produced by reptarena- and hartmanivirus superinfected I/1Ki-Δ (*B. constrictor*, kidney) and V/2Hz-Δ (*B. constrictor*, heart) cells permanently infected with SDeV. Briefly, we collected the supernatants up to 14 dpi, cleared by centrifugation (30 min, 4,200 × *g*, +4°C), and filtered through a 0.45-μm syringe filter (Millipore). The supernatants were placed in either 25- by 89-mm (for SW28 rotor) or 14- by 89-mm (for SW41 rotor) Ultra-Clear tubes (Beckman Coulter) that had been underlaid with 3 ml (for SW28 rotor) or 1 ml (for SW41 rotor) of 25% (wt/vol) sucrose (in TBS) using a thin needle. Ultracentrifugation (27,000 rpm,1.5 to 2 h, +4°C, for both SW28 and SW41 rotor) was performed, the supernatant and sucrose cushion were poured off, and the pellets were resuspended in TBS. We used the same protocol for concentrating the supernatants collected from transfected I/1Ki-Δ cells. For density gradient ultracentrifugation, we used Gradient Master (BioComp) to prepare either 10 to 70% (first two attempts) or 20 to 55% (third attempt) linear sucrose gradients (in TBS) in 14- by 89-mm (for SW41 rotor) Ultra-Clear tubes (Beckman Coulter) following the manufacturer’s protocol. We loaded the viruses concentrated as described above on top of the gradient, performed ultracentrifugation (40,000 rpm for either 18 h [first attempt], 4 h [second attempt], or 1.5 h [third attempt], +4°C, SW41 rotor, Beckman Coulter), and collected approximately 600-μl fractions by puncturing the tubes with a thin (23-gauge [23G] or 25G) needle.

### Electron microscopy.

We prepared a sample of the material pelleted for the third density gradient ultracentrifugation for electron microscopy, and used 4 μl (corresponding to roughly 1.5 ml of nonconcentrated supernatant) of supernatant from mock, UGV-1 or HISV-1 superinfected I/1Ki-Δ cells and UGV-1 or HISV-1 superinfected V/2Hz-Δ cells. We applied the samples to glow discharged grids by using the side blotting method described by Scarff et al. (62); after 45-s adsorption, we washed the grids by immersing the carbon surface of the grids twice in Milli-Q water droplets before staining with 2% phosphotungstic acid at neutral pH for 30 s. A JEOL JEM-1400 transmission electron microscope was used for image acquisition at ×200,000 magnification.

### Virus titration.

To determine whether superinfections or transfections with viral glycoproteins had induced formation of infectious SDeV particles, we performed 10-fold dilution series of the supernatants and used the diluted supernatants to inoculate uninfected I/1Ki cells. At 4 to 6 dpi, the cells were fixed and stained for SDAg as described above. The number of fluorescent focus-forming units (fFFU) was determined by enumerating the number of SDAg-positive cells using fluorescence microscopy. In addition, we quantified the number of fluorescent cells from the plates used for titrating the supernatants from superinfected or glycoprotein-transfected cells using Opera Phenix High Content Screening System (PerkinElmer), a method provided by FIMM (Institute for Molecular Medicine Finland) High Content Imaging and Analysis (FIMM-HCA). Each dilution was represented by two or three parallel wells, and when possible, two consecutive dilutions were used for calculating the number of fFFU in the original sample.
