# Supplementary figures and images for: Snake Deltavirus Utilizes Envelope Proteins of Different Viruses To Generate Infectious Particles
Source: mBio. 2020 Mar 17;11(2):e03250-19. doi: 10.1128/mBio.03250-19 (PMC7078484; doi:10.1128/mBio.03250-19)

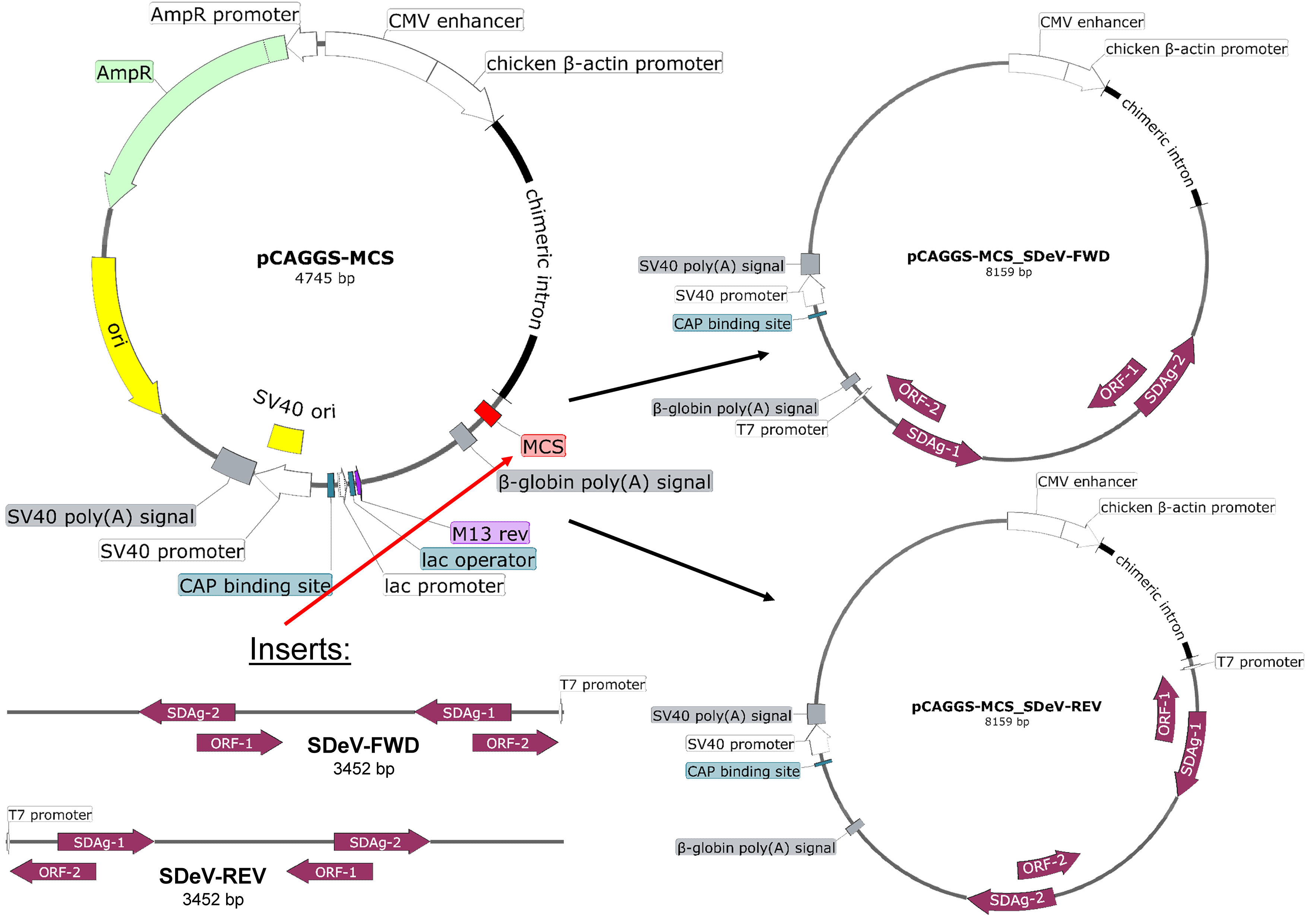

Supplement: FIG S1 [file mBio.03250-19-sf001.tif]

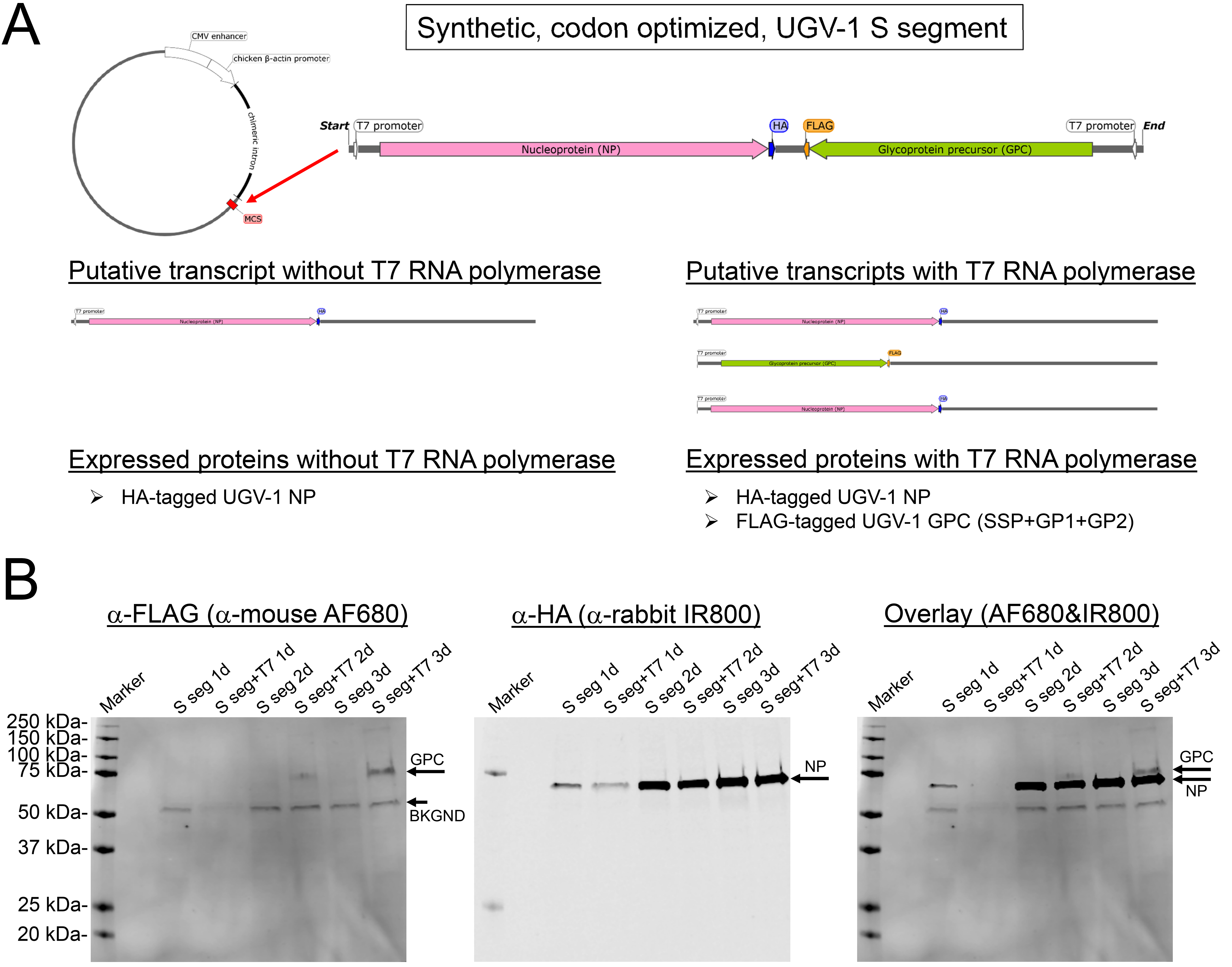

Supplement: FIG S2 [file mBio.03250-19-sf002.tif]

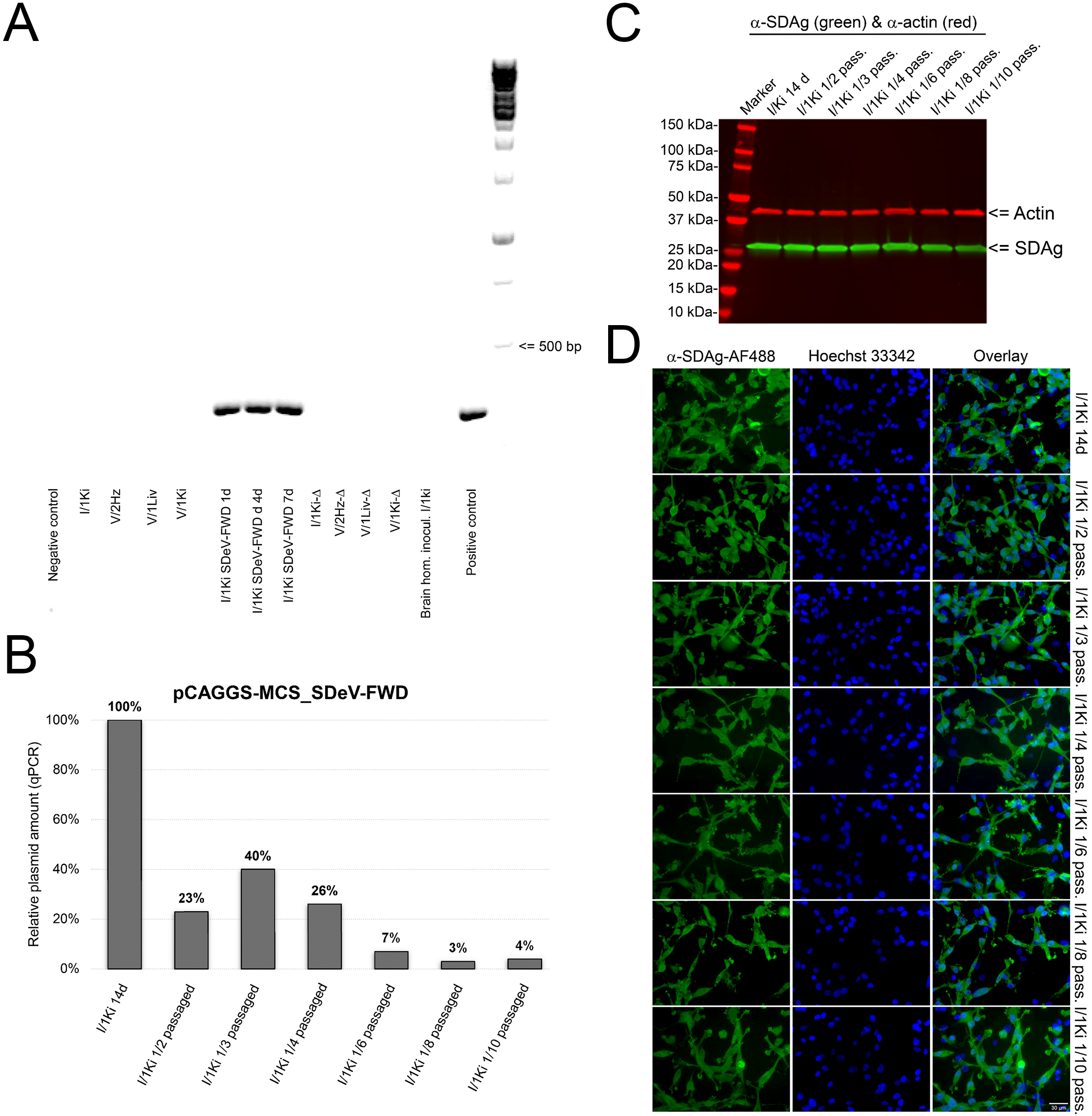

Supplement: FIG S3 [file mBio.03250-19-sf003.tif]

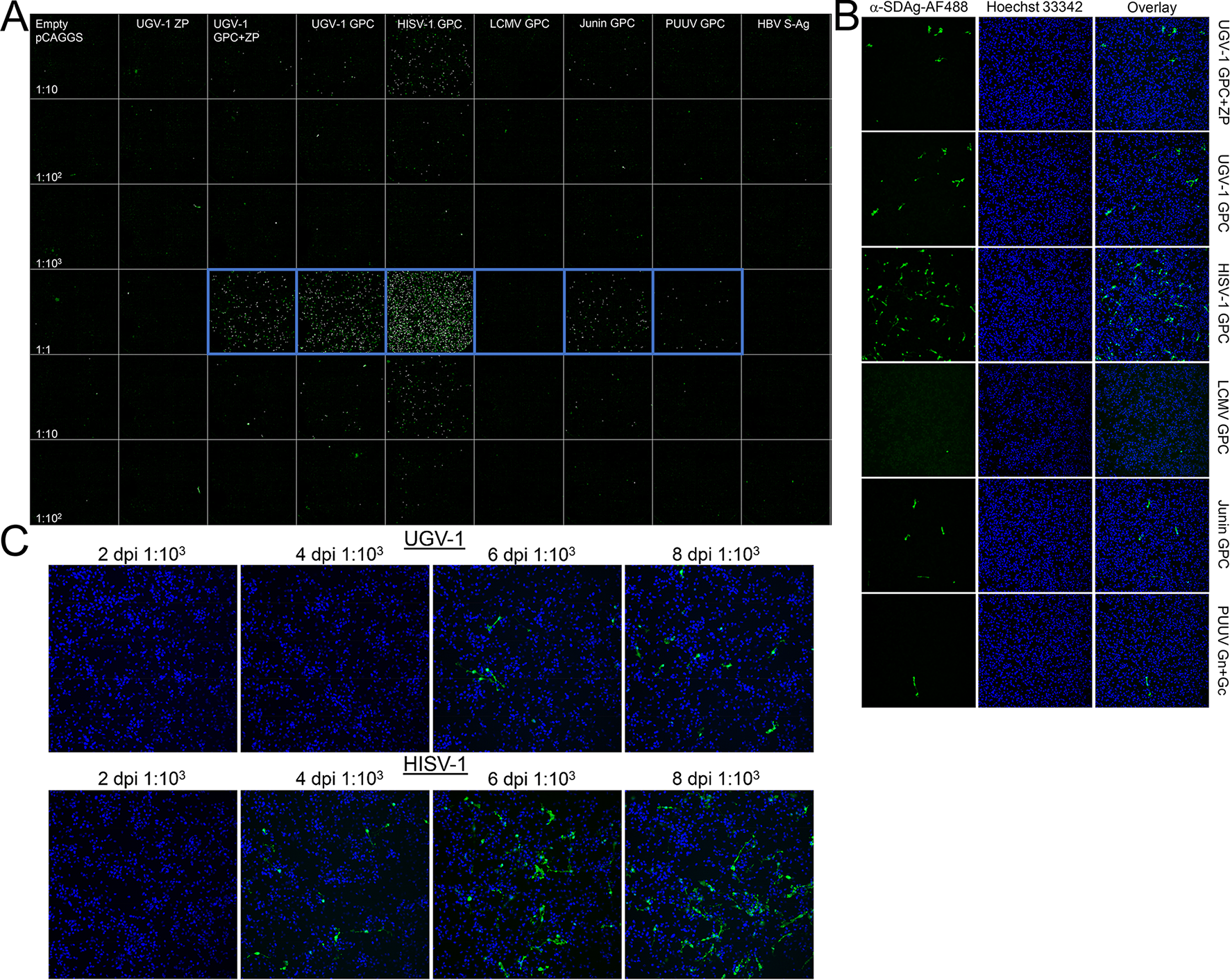

Supplement: FIG S4 [file mBio.03250-19-sf004.tif]
